# Prevalence of Vestibular Dysfunction in Children With Neurological Disabilities: A Systematic Review

**DOI:** 10.3389/fneur.2019.01294

**Published:** 2019-12-17

**Authors:** Shashank Ghai, Mireille Hakim, Elizabeth Dannenbaum, Anouk Lamontagne

**Affiliations:** ^1^School of Physical and Occupational Therapy, McGill University, Montreal, QC, Canada; ^2^Feil and Oberfeld Research Centre of the Jewish Rehabilitation Hospital: Centre for Interdisciplinary Research of Greater Montreal (CRIR), Laval, QC, Canada; ^3^Concordia Physio Sport, Laval, QC, Canada

**Keywords:** balance, pediatrics, congenital disease, neurodevelopmental disorder, vestibular system, prevalence

## Abstract

**Background:** In children with neurological or neurodevelopmental conditions, vestibular disorders may co-exist with the primary condition and further contribute to disability and restriction in functional independence and participation. Awareness of their existence may favor an early diagnosis and better treatment outcomes.

**Objectives:** To determine the prevalence of vestibular dysfunction in children and adolescents (3–21 years old) diagnosed with either cerebral palsy (CP), traumatic brain injury (TBI), sensorineural hearing loss (SNHL), or cochlear implantations (CI).

**Methods:** Four researchers systematically reviewed the literature from three databases (EMBASE, MEDLINE, CINAHL) until June 2018.

**Results:** Twenty-four studies were analyzed in this systematic review. A single, high-quality study reports a prevalence of 48.4% of spastic CP children having a saccular dysfunction. Three fair-quality studies report a prevalence of 14.6–81%, 21 days post-TBI. Twelve poor-to-high quality studies demonstrate a prevalence of 18.7–96.1% in children with SNHL. A prevalence range of 3–84% in children with CI is reported by nine fair-to-high quality studies.

**Conclusion:** Clinicians should be aware of the prevalence of vestibular dysfunction in these populations and implement appropriate assessments to improve treatment outcomes.

## Introduction

The vestibular system is involved in key functions such as gaze stabilization, balance, postural orientation, and spatial navigation (1, 2). Thus, a dysfunction of the vestibular system can be very debilitating, hindering the completion of activities of daily living and necessitating medical attention (3, 4). Across the different studies conducted in the adult population, the prevalence of vestibular disorders is relatively well-documented using either vestibular symptoms such as vertigo, balance tests or the prevalence of specific vestibular disorders (5, 6). In fact, vestibular disorders would affect almost 6.5–7.4% of individuals in their adulthood (7, 8), with a larger prevalence in older adults (7). The prevalence of vestibular disorders in childhood, however, is not as well documented (9).

The American Psychiatric Association states that vestibular hypofunction would present in children as poor balance leading to falls, especially during high-level motor skills such as hopping, skipping, or walking on a balance beam (10). In babies, it would translate as delayed sitting, standing and walking. Collectively, studies on vestibular function in childhood indicate that vestibular disorders do occur in children and that these may lead to signs and symptoms of vertigo, dizziness, decreased balance, and impaired vestibulo-ocular reflex (VOR) (11–15). In presence of neurological or neurodevelopmental disorders, as typically seen pediatric neurorehabilitation, the prevalence of vestibular disorders could further increase due to the vestibular system's connections to and from the central and peripheral nervous system. If untreated, future complications might arise and hinder the child's ability to recover or acquire functional independence that is essential for full participation in society (16). Thus, there is a need to document the prevalence and nature of peripheral vestibular dysfunction as a comorbidity, or as a consequence of a neurological or neurodevelopmental condition. Moreover, clinicians often rely on parent's inputs to screen for vestibular dysfunction, especially when the child is too young to verbalize their symptoms (17). Having the clinical community aware of the prevalence of vestibular dysfunctions in children will increase the likelihood of them being assessed and treated (9).

The purpose of this systematic review is to fill an important gap in knowledge on the prevalence of vestibular disorders in children with neurological and neurodevelopmental disorders worldwide. A recent review by Van Hecke et al. (18) reported the prevalence of vestibular dysfunction in children with autism spectrum disorder, attention deficit hyperactivity disorder, intellectual disability disorder and specific learning disorder. However, the authors did not analyze the prevalence in other common neurodevelopmental disabilities. The following four conditions have been selected in the present study due to their high prevalence among the pediatric rehabilitation population and indications in the literature of symptoms possibly associated with vestibular disorders:

**Sensorineural hearing loss** (SNHL) is a congenital or acquired hearing impairment resulting from a defect in one (unilateral) or both (bilateral) cochlea, auditory receptors, or auditory nerves and their subsequent connection to the brain (19, 20). The vestibular end organs are anatomically connected to the cochlea and share the same origin embryologically. Therefore, children suffering from SNHL may exhibit abnormalities of the vestibular system (21, 22). Almost, 20 to 70% of children with SNHL present with an element of vestibular end-organ dysfunction (22). Some authors report that the prevalence of vestibular dysfunction in children with SNHL can go up to 85% of patients (22, 23). Nevertheless, it is important to further investigate the prevalence of peripheral vestibular dysfunction in children with SNHL in order to provide them early access to rehabilitation and prevent complications.

**Cochlear implantation (CI)** is a procedure done in children with severe-to-profound SNHL to improve their hearing and help with language acquisition during childhood (13, 24, 25). CI may hurt the vestibular system by directly damaging the inner ear structures from the passage of the electrodes into the scala tympani, or from inflammation, fibrosis, ossification, or endolymph hydrops in the inner ear due the post-surgical healing or body's reaction to the implant (13, 26). Furthermore, the saccule is the structure that is most likely to be damaged regardless of the surgical approach used (27). Since vestibular function is important for the development of motor milestones, clinicians must be aware of the prevalence of peripheral vestibular dysfunction and integrate vestibular rehabilitation to the treatment when necessary.

**Cerebral palsy and pediatric stroke** (herein referred to as CP for both conditions) result from a brain lesion before, at or after birth which causes movement disorders (weakness, poor coordination, ataxia), poor balance and posture (28), and abnormal muscle tone (hypo or hypertonia). It can present as hemiplegia, diplegia, or quadriplegia (29). It is one of the most common causes of physical disability in early childhood, occurring in 2/1,000 live births (30). Due to the large span of potential brain involvement occurring in every presentation of CP, and to the sensory impairments proven to result from it (31), the central vestibular system might be compromised. In addition, decreased utilization of the vestibular end-organs due to compromised motor abilities might result in their underdevelopment (32). Clinicians should be aware of the prevalence of these disorders to decrease their impact on the patient's prognosis and to maximize their potential for acquiring function.

**Traumatic brain injury (TBI)** is the most common acquired neurological disorder in the pediatric population (33). During the impact of trauma, both the central and peripheral vestibular systems are vulnerable to injury from the translated force (34). Signs and symptoms related to vestibular dysfunction such as dizziness, postural instability, benign paroxysmal positional vertigo (BPPV), altered VOR as well as deficits in balance and sensory integration have been reported in children even years following the trauma (35). Hence, awareness of these sequelae, appropriate vestibular assessment, and pertinent treatment strategies are crucial for the rehabilitation of a patient post TBI, regardless of the severity of the injury.

Specifically, the main objective of this systematic review was to investigate the prevalence of vestibular dysfunction in children and adolescents aged 3–21 years with CP, TBI, SNHL, and CI. The secondary objective was to provide more information in terms of the nature of vestibular dysfunctions observed, tests that were employed and possible gaps in knowledge for specific subpopulations included in this study. The anticipated impact of this study is to raise awareness of vestibular dysfunctions for rehabilitation specialists and healthcare professionals, in order to lead to better patient-centered care and optimal rehabilitation outcomes.

## Methods

This review was carried according to guidelines mentioned in Preferred Reporting Items for Systematic Reviews and Meta-analysis statement (PRISMA) (36). A PRISMA checklist has been provided as Supplementary Table 3.

### Search Strategy and Inclusion Criteria

Three online scientific databases were used to conduct a systematic search of the literature: OVID MEDLINE (Medical Literature Analysis and Retrieval System Online: 1946 to June 26th, 2018), CINAHL (Cumulative Index to Nursing and Allied Health Literature: 1982 to June 26th, 2018), and OVID EMBASE (Excerpta Medica Database: 1946 to June 26th, 2018). A meticulous search while using Medical Subject Headings (MeSH) keywords was performed on these databases. A sample search strategy on EMBASE database has been provided as Supplementary Table 1. A “Pediatric” and an “Observational Studies” pre-set filters were included to limit the searches to the 0–21 population, and to focus on observational studies, respectively. In addition to the database searches, cross-referencing and hand-searches were performed, and relevant studies were included in the search results.

**Figure 1:**
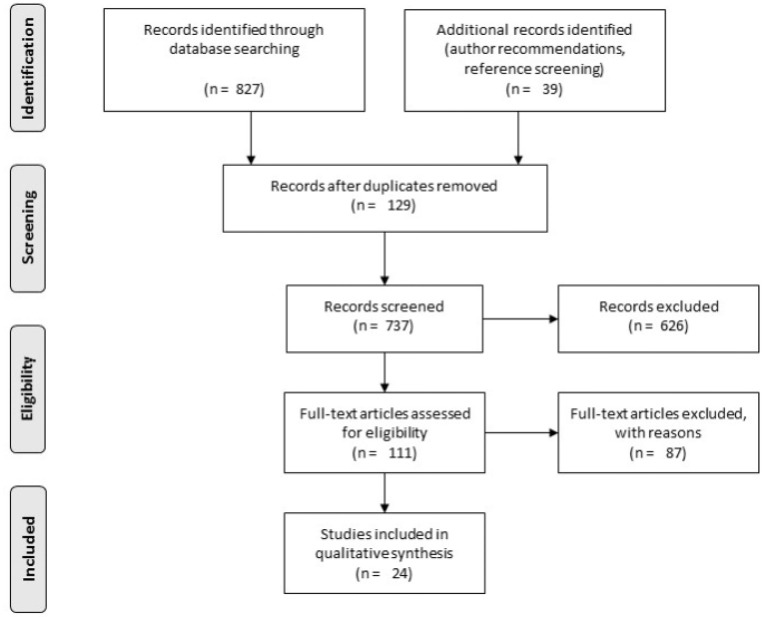
Illustrates the PRISMA search strategy.

From the three database searches, 737 studies were screened by title and abstract independently by four reviewers. Two reviewers screened half of the studies, and two reviewers the other half. Through the same screening method, 111 studies were then assessed by full text for eligibility, and 87 were excluded as per the criteria discussed below. Twenty-four studies were finally included in the review (Figure 1). Pairs of reviewers assigned to the same studies met regularly, and, if a disagreement took place, a third reviewer from the other pair was consulted. The following inclusion criteria were used to include the studies screened in the analysis: (1) Observational studies: cohort, cross-sectional, or case-control studies; (2) Studies that focus on children and adolescent aged 3–21 years old, from any country; (3) Studies that focus on children who belong to 1 or more of the 5 populations under study: CP, SNHL, CI, and TBI; (4) Studies looking at any vestibular dysfunction, diagnosed through at least one standardized vestibular test; (5) Studies that declare a prevalence of vestibular dysfunction, or from which a prevalence can be calculated. Here, the prevalence was defined as the proportion of population demonstrating a similar characteristic during a given period of time (37); (6) Studies written in English of French.

### Quality Assessment

The methodological quality of the studies was evaluated using subsets of the Newcastle-Ottawa Scale (NCO scale), a star-based system in which a study assessed receives a star if the described characteristic is met (38). Cross-sectional studies can receive a maximum of 10 stars, whereas the maximum score for Case-Control and Cohort Studies is 9. For both types of study design, scores of <4 stars were considered as reflecting a study of poor quality, 4–6 stars as fair quality and >6 stars as high quality (39), while considering the total score. The initial agreement among the pair of reviewers in charge of appraising a study was calculated using Cohen's kappa. As applicable, discrepancies in scores were discussed and an agreement was reached for each of the studies.

### Data Extraction

Twenty-four selected studies were independently appraised and reviewed. Pertinent information of each study was extracted and organized on a data extraction table (Tables 1–**3**). Following characteristics of each study was mentioned in the table:

1. Study type and sample size.
2. Descriptive information about the subjects assessed: age, sex, condition, severity of condition, and comorbidities.
3. The presence and descriptive information of a control group composed by healthy children.
4. Outcome measures: outcomes (type and severity), outcome assessment methods (tools and tests).
5. Prevalence of vestibular dysfunction reported by the authors.
6. Quality of each study assessed according to the NCO scale.

**Table 1:** Results of quality assessment of the studies using the Newcastle—Ottawa Assessment Scale (According to, Newcastle Ottawa Quality assessment forms, observational studies can receive a maximum of 10 stars, whereas cohort and case-control studies can be attributed a maximum of 9 stars).

**References**	**Study design**	**Selection**	**Comparability**	**Outcome/ exposure**	**Total Score**
Sokolov et al. (40)	Cohort	^*^,0,^*^,^*^	^*^,0	^*^,0,0	5/9
Raj and Gupta (21)	Cross sectional	0,^*^,0,^**^	^*^,^*^	^**^,0	7/10
Devroede et al. (41)	Cohort	^*^,^*^,0,0	^*^,^*^	^**^,^*^	7/9
Corwin et al. (42)	Cohort	^*^,0^*^,0	^*^,0	^*^,^*^,^*^	6/9
Thierry et al. (24)	Cohort	0,0,^*^,^*^	^*^,0	^*^,0,0	4/9
Wolter et al. (43)	Case control	^*^,0,^*^,^*^	^*^,0	^*^,^*^,0	6/9
Akbarfahimi et al. (44)	Case-Control	^*^,^*^,^*^,^*^	^*^,^*^	^*^,^*^,^*^	9/9
Mucha et al. (45)	Cross sectional	0,^*^,0,^*^^*^	0,0	^*^^*^,0	5/10
Cushing et al. (46)	Cross sectional	^*^,^*^,^*^,^*^^*^	^*^,^*^	^*^^*^,^*^	10/10
Schwab and Kontorinis (47)	Case control	^*^,0,0,0	^*^,0	^*^,^*^,0	4/9
Jafari and Asad Malayeri (48)	Case Control	^*^,^*^,^*^,^*^	^*^,^*^	^*^,0,^*^^*^^*^	8/9
Zhou et al. (19)	Cohort	0,0,^*^,0	^*^,0	^*^,0,0	3/9
Licameli et al. (13)	Cohort	0,^*^,^*^,^*^	^*^,^*^	^*^,^*^,^*^	8/9
Jacot et al. (25)	Cohort	^*^,^*^,^*^,0	^*^,^*^	^*^,^*^,0	7/9
Cushing et al. (22)	Cross sectional	^*^,^*^,^*^,^*^^*^	^*^,^*^	^*^^*^,^*^	10/10
Shinjo et al. (49)	Cross sectional	0,0,0,^*^^*^	^*^,^*^	^*^^*^,0	6/10
Jin et al. (50)	Cross sectional	^*^,^*^,0,^*^^*^	^*^,^*^	^*^^*^,^*^	9/10
Bouccara et al. (51)	Cohort	^*^,0,^*^,0	^*^,0	^*^,^*^,0	5/9
Lisboa et al. (15)	Cohort	^*^,0,^*^,^*^	^*^,0	^*^,^*^,^*^	7/9
Rine et al. (20)	Cross sectional	0,0,0,^*^^*^	^*^,0	^*^^*^,0	5/10
Horak et al. (52)	Case Control	^*^,^*^,^*^,0	^*^,^*^	^*^0,0,^*^	7/9
Vartiainen et al. (34)	Cohort	^*^,0,^*^,0	^*^,^*^	^*^,^*^,0	6/9
Potter and Silverman (53)	Case Control	^*^, 0, 0, ^*^	^*^,0	^*^,0,0	4/9
Rosenblüt et al. (54)	Case control	^*^,0,^*^,^*^	^*^,0	^*^,^*^,0	6/9

Specific reasoning for excluding the studies from our review has also been provided in Supplementary Table 2.

### Data Analysis and Categorization of Study

The overall percentages of prevalence of vestibular dysfunction indicated in each study were extracted and a range of percentage of dysfunction per population was found. Where available, percentages of specific types of dysfunction and percentages found through different assessment tools were also noted, and categories were created in order to emphasize the most common type of dysfunction and assessment tool of each population.

**Figure 2:**
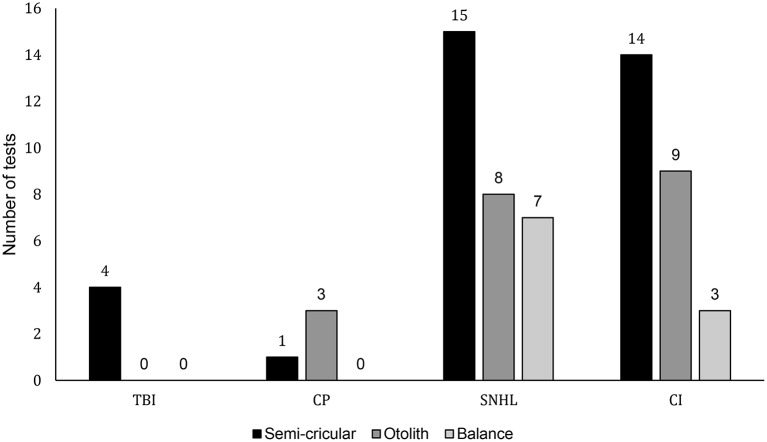
Illustrates the frequency of usage of assessment tools per condition. The y axis indicated the number of time a given type of assessment (for semi-circular canals: rotary chair, caloric, vHIT, VOMs, VOR, VNG, otolith: bucket test, VEMP, or balance: SOT, BOT2, Peabody test) was used while the x axis indicates the different pediatric conditions included in the study and respective number of studies in parenthesis. Conditions read as follows: TBI, Traumatic brain injury; CP, cerebral palsy; SNHL, sensorineural hearing loss; CI, cochlear implant.

In pediatric clinical practice, it is of utmost importance to choose assessments tool that shows good psychometric properties, but which can also be easily administered to children. We included reliable and valid standardized vestibular assessment tools (55). For ease of review, the different assessments were grouped depending on whether they address the labyrinthine (semicircular canal) function, otolith function, or integrated balance (see Figure 2).

## Results

### Study Selection

Figure 1 illustrates the study-selection process in this review through the PRISMA flowchart (36). The overall search yielded 827 studies from the three databases, and 39 from the hand search. Among the 866 studies, 129 duplicates were removed using EndNote 7X, and 826 were excluded based on title and abstract screening. Moreover, 86 studies were excluded after full-text screening. Appendix B contains these studies and their reason for exclusion. A total of 24 studies were included and underwent the data extraction and appraisal procedure. Among these 24 studies, 12 focused on children with SNHL, 8 on children post-CI, 3 on children with TBI, and 1 on children with CP.

### Study Characteristics

Characteristics of the different studies in terms of their country origin, study design, participants, etiology, and severity of the primary condition (e.g., of CI, CP, etc.), comorbidities, vestibular assessment tools employed, and main results can be found in Table 1 (Peripheral Neurological Conditions: SNHL and CI), and Table 2 (Central Neurological Conditions: CP and TBI). Among the 24 selected studies, there were 10 cohort studies, 7 cross-sectional studies, and 7 case-control studies. Sample sizes for the population of interest varied across the different studies, ranging from 20 to 261 for CI and SNHL and 64–247 for TBI. The unique studies for CP had sample sizes of 31. Participants' ages from the included studies ranged from 7 months to 21 years old. Only in 2 of the 24 studies the participant's presented with a comorbidity, that is a documented condition other than the primary diagnosis (20, 54). Eleven studies took place in North America (Canada and the United States), 6 in Europe (3 in France, 1 in Belgium, 1 in Finland, and 1 in Germany), 5 in Asia (3 in India, 2 in Japan), 1 in the Middle-East (Iran), and 1 in South America (Brazil).

**Table 2:** Studies addressing central neurological conditions (Sensorineural hearing loss and cochlear implant).

**References**	**Country**	**Study design**	**Participants**	**Age**	**Etiology and severity**	**Comorbidities**	**Vestibular assessment tools**	**Main results, outcomes and prevalence**	**NCO score**
Sokolov et al. (40)	Canada	Cohort	20 children with unilateral SNHL	Mean age: 8.8 y.o.	Unilateral SNHL; severe-profound SNHL (PTA = 96 dB), moderate-severe SNHL (PTA = 67 dB); mild-moderate SNHL	None specified	Caloric test vHIT cVEMP oVEMP	Abnormal vestibular function found in 12/20 (60%) patients. Abnormal utricle response found in 4/12 (33%) of cases, through oVEMP testing. Abnormal saccular response absents in 3/18 (17%) of cases, through cVEMP testing. Overall otolothic dysfunction shown in 4/19 (21%) patients through either cVEMP and/or oVEMP testing. Abnormal horizontal canal function observed in 7/20 (35%) cases through vHIT testing and observed in 9/19 (48%) cases through calorictesting.	5/9
Raj and Gupta (21)	India	Cross sectional	50 children with SNHL	Mean age: 5.48 y.o. Age range: 4–9y.o	Congenital profound and severe SNHL	None reported	Warm air caloric test	Abnormal vestibular function found in 9/48 (18.75%) cases through caloric testing	7/10
Thierry et al. (24)	France	Cohort, retrospective	43 children with unilateral CI	Mean age: 2.9 y.o. Age range: 6–15.1y.o.	Etiology of SNHL: genetic mutations, infections, Waardenburg syndrome, meningitis, Kallman syndrome, or idiopathic	None specified	HIT Bithermal caloric test VEMP	Decreased ipsilateral vestibular function post-CI observed in 8/43 (18.6%) children. Abnormal contralateral vestibular function found in 3/43 (7%) cases. Worsening of vestibular function post-CI experienced by 2/12 (16.7&) patients. Improvement of vestibular function found in 4/12 (4/12)cases.	4/9
Wolter et al. (43)	Canada	Cohort, retrospective	187 Group 1: 22 children who experienced CI failure Group 2: 165 children who did not experience CIfailure	Not specified	Etiology of SNHL: Meningitis, cochleovestibular anomaly, Usher syndrome, Connexin-26 mutation, Cytomegalovirus	None specified	Bithermal caloric test Rotational head impulse test/vHIT VEMP	Abnormal horizontal canal function found in 18/22 (81.8%) children with CI failure vs. 78/165 (47.3%) children without CI failure through bithermal caloric testing. Abnormal high frequency horizontal canal function found in 16/22 (72.2%) children with CI failure vs. 57/165 (34.5%) of children without CI failure through vHIT and/or high frequency rotational chair testing. Abnormal saccular function observed in 18/22 (81.8%) children with CI failure vs. 76/165 (46.1%) children without failure through VEMPtesting.	6/9
Devroede et al. (41)	Belgium	Cohort, Retrospective	26 children with unilateral CI, before and after	Mean age: 6.75 y.o Age range: 1–13y.o	SNHL as part of a clinical syndrome, genetic mutations, post meningitis, CMV infection, auditory neuropathy spectrum disorder, or idiopathic ± unilateral CI ± bilateral /contralateral CI	None reported	Caloric test VEMP	Pre-contralateral implantation, 2/26 (8%) showed bilateral areflexia, 16/26 (61%) showed hyporeflexia (i.e., 69% presented with hyporeflexia). Otholitic functioning was abnormal in 5/24 (21%) patients' pre-contralateral implantation, and in 9/24 (37%) post-contralateral implantation, as recorded through VEMP Horizontal canal function changed in 32% of the patients tested through caloric stimulation	7/9
Cushing et al. (46)	Canada	Cross sectional	Children Group 1: 119 children with unilateral CIs Group 2: 34 childrenpre-CI	Mean age: 12.95 y.o Age range: 3.6–20y.o	Profound SNHL with unilateral CI or before implantation procedure	None reported	Caloric test Rotatory chair test VEMP VOR	Abnormal horizontal canal function found in: 69/139 (50%) through caloric testing, of which 18/69 (26%) reflect mild to moderate unilateral abnormalities, and 51/139 (37%) severe hypofunction or areflexia. Abnormal horizontal canal function found in: 64/139 (47%) through rotatory chair testing. Bilateral reduction in VOR seen in 29% (40/139) Absent saccular function bilaterally in 32/135 (21%) and unilaterally in 40/135 (30%) through VEMP. All children with meningitis (*n* = 11) and 46% with radiologic cochleovestibular anomalies (*n* = 31) had horizontal canal dysfunction, whereas 45 and 46%, respectively, displayed saccular dysfunction.	10/10
Schwab and Kontorinis (47)	Germany	Case control	Group 1: 40 children with SNHL Group 2l: 40 normal-hearingchildren	Age range: 4–20 y.o,	Deaf of hearing -impaired children admitted for CI exam	None specified	Caloric test SOT, MCT,ADT	Abnormal vestibular function found in 16/33 (40%) cases through caloric testing. Hypoexcitabililty of vestibular function found in 27/66 (41%) tested ears, whereas hyperexcitability found in 2/66 (3%) tested ears	4/9
Jafari and Asad Malayeri (48)	Iran	Case control	Group 1: 30 children with SNHL Group 2: 30 healthychildren	Group 1: Mean age: 6.93 y.o Age range: 6–9 y.o Group 2: Mean age: 7.18 y.o Age range: 6–9y.o	SNHL congenital or early acquired bilateral profound SNHL	None specified	VEMP, ABR, BOT-2, balance subtest	Abnormal vestibular function was found in 28/32 (87.5%) ears tested through VEMP. Asymmetrical vestibular response found in 4/30 (13.3%) cases through VEMP testing. No vestibular response found in 12/30 (40%) children through VEMPtesting.	8/9
Licameli et al. (13)	USA	Cohort	Group 1: 42 children with unilateral CI Group 2: 19 children pre andpost-CI	Group 1: Mean age: 9 y.o Age range: 5–22 y.o Group 2: Mean age: 8 y.o Age range: 2–23y.o	Patients in Group 1 being considered for a second CI on the contralateral side.	None reported	VOR Computerized dynamic posturography,VEMP	60% of all patients had abnormal finding(s) in at least one laboratory test. Abnormal ipsilateral VOR response observed in 22/42 (52%) of Group 1. Abnormal findings on Computerized dynamic posturography testing found on 15/38 (39%) of Group 1, which indicate peripheral vestibular weakness and/or sensory organization deficit. Reduced or absent VEMP responses found on 12/15 (80%) of Group 1. Pre-CI, 2/19 (10%) of group 2 patients did not present any VEMP response. Post-CI, 16/19 (84%) of group 2 patients indicated disappearances or reduction of VEMP responses (elevation in VEMP thresholds and/or decrease in VEMPamplitudes).	7/9
Zhou et al. (19)	USA	Cohort, retrospective	Group 1: 23 children with bilateral SNHL Group 2: 12 healthy children	Group 1: Age range: 2–16 y.o. Group 2: Age range: 4–18y.o	SNHL: Moderate, severe, profound. SNHL etiology: bialletic GJB2 mutation, congenital cytomegalovirus infection, bacterial meningitis, cogan syndrome	None specified	VEMP	Abnormal saccular function found in 21/23 (91.3%) through VEMP testing.	3/9
Jacot et al. (25)	France	Cohort, Prospective & retrospective	Children with SNHL, 89 of which participated after CI procedure	First examination: Mean age: 51 mon Age range: 7 mon−16.5 y.o Second examination: Mean age of 52.8 mon Age range: 7 mon−12y.o	SNHL—to be implanted with CI Unilateral CI	None reported	Bi-caloric test Earth vertical axis rotation Off vertical axis rotation VEMP	Abnormal bilateral vestibular function found in 112/224 (50%), 45/224 (20%) showed complete areflexia, 50/224 (22.5%) showed partial asymmetrical hypo-excitability, and 17/224 (7.5%) showed partial symmetrical hypo-excitability. Changes of vestibular function post-CI found in 51/71 (71%), from which 7/70 (10%) acquired ipsilateral areflexia Long-term follow up reports partial recovery of vestibular responses is observed in 18.5% of the cases,post-CI	7/9
Cushing et al. (22)	Canada	Cross sectional	Children with unilateral CIs	Mean age: 3–19.3 y.o	Severe to profound SNHL with unilateral cochlear implants	None reported	Caloric test Rotatory chair test VEMP BOT-2	Abnormal horizontal canal function found in: 16/32 (50%) through caloric testing, and 14/37 (38%) through rotatory chair testing. Absent saccular function bilaterally in 5/26 (19%) and unilaterally in 5/26 (19%) through VEMP. Mean BOT-2 scores for children with SNHL and CI were significantly poorer than the norm.	10/10
Shinjo et al. (49)	Japan	Cross sectional	Children with SNHL	Mean age: 54.2 mon Age range: 31–97 mon (2.5–8y.o)	Conditions included: Severe SNHL, fitted with hearing aids, congenital profound SNHL, progressive hearing impairment, LVA Etiology: infection, meningitis, congenital auditory nerve disease, common cavity malformation in the inner ear, cochlear nervemalformation	None specified	Ice water caloric test Damped-rotational chair test VEMP	Abnormal responses in at least 1 test found in 85% of children Asymmetrical canal responses found in 7/20 (35%) cases, hypo-reactions found in 2/20 (10%) cases, and absence of response observed in 8/20 (40%) cases, all through caloric testing. Decreased uni-directional canal response observed in 1/20 (5%) cases, decreased bidirectional response observed in 2/20 (10%) cases, and absence of response observed in 3/20 (15%) cases, all through rotational chair testing. Asymmetrical saccular response found in 6/20 (30%) cases, and absence of bilateral response found in 4/20 (20%) cases, all through VEMP testing.	6/9
Jin et al. (50)	Japan	Cross sectional	Group 1: 12 children who underwent CI surgery Group 2: 9 healthychildren	Group 1: Mean age: 3.8y.o Age range: 2–7 y.o Group 2: Age range: 8 mon−10y.o	Cochlear implantation (CI)	None	VEMP Calorictest	Semicircular canal hypofunction found through ice water caloric testing in 6/10 (60%) of cases, and areflexia on 4/10 (40%), post implantation.Saccular function reduction observed in 7/12 (58.3%) of patients, through VEMP testing, post-implantation.	9/10
Bouccara et al. (51)	France	Cohort, Prospective	Children Group 1: 240 childrenpost-CI Group 2: 28 children assessedpre-CI	Mean age: 7.5 y.o Age range: 2–15y.o	Idiopathic, genetic, or drug-related hearing loss	None reported	VNG	9/268 children (3%) present with abnormalities as per the VNG assessment at some point after the implantation	5/9
Lisboa et al. (15)	Brazil	Cohort	Children with SNHL	Age range: 10–14 y.o	Severity of disease ranged from profound /severe bilateral to unilateral hearing loss	None reported	Ocular and labyrinthic tests Caloric Test RotatoryTest	Alterations on caloric testing found in 25/26 (96.1%) patients, from which: Unilateral hyporreflexia was found in 4/26 (15.3%) patients Bilateral hyporreflexia was found in 20/26 (76.9%) patients Directional preference of asymmetrical nystagmus was found in 1/26 (3.8%)patients	7/9
Rine et al. (20)	USA	Cross sectional	Children with SNHL	Age range: 26–83 mon (2–6.9 y.o)	Profound bilateral hearing loss	Developmental delay	PDMS, SCPNT	Hypoactive vestibular function found in 20/39 (51.3%) cases through SCPNT. Hyperactive vestibular function found in 18/39 (46.2%) cases through SCPNT. Children with moderate to profound sensorineural hearing loss have a delay in gross motor development which is progressive and related to vestibular hypofunction.	7/10
Horak et al. (52)	USA	Case-control	Group 1: 54 normal developing children Group 2: 30 children with bilateral hearing impairment Group 3: 15 children with learning disabilities	Age range: 7–12 y.o. Mean age: 9.2y.o	Bilateral hearing loss acquired within the first two years of life, congenital, post-meningitis, unknown etiology	None reported	Horizontal VOR Sensory organization for postural orientation test BOT for MotorProficiency	Abnormal VOR observed in 20/30 (67%) patients. Hearing-impaired children with vestibular loss scored at the 29th percentile in motor proficiency because of a mean balance score only half thenormal.	7/9
Potter and Silverman (53)	USA	Case Control	Children with SNHL	Mean age: 6.1 y.o Age range: 5–8.11y.o.	Hearing loss in the better ear ranged from 55 to 120 dB. Average hearing loss: 100.5dB.	None reported	SCPNT Standing Balance subtests (eyes open and closed) of the Southern California Sensory Integrationtests	Abnormal (hypoactive) vestibular response found in 20/34 (58.8%) of cases through rotatory test (scores compared to norms). 15/34 (44.1%) showed no response to vestibular stimulation. With eyes open, 44.1% of the deaf children had abnormal standing balance. With eyes closed, 35.3% had abnormalbalance.	4/9
Rosenblut et al. (54)	USA	Case Control	Group 1: 107 children with SNHL Group 2: 57 aphasic children (not relevant) Group 3: 16 healthy children	Age range: 3–13 y.o	SNHL resulting from: Meningitis family history, maternal rubella, complications during pregnancy, or congenital brain abnormality	Possible congenital brain abnormality	Nystagmus assessment through modification of the test originated by Fitzgerald and Hallpike	Depressed vestibular function found in 25/107 (23.4%) cases, and absent response reported in 27/107 (25.2%) according to nystagmus assessment.	5/9

*vHIT, video head impulse test; cVEMP, cervical vestibular evoked myogenic potential; oVEMP, ocular vestibular evoked myogenic potential; SOT, sensory organization test; MCT, motor control test; ADT, adaptation test; ABR, auditory brainstem response; BOT-2, Bruininks-oseretsky test (second edition); LVA, large vestibular aqueduct; VNG, Videonystagmography; PDMS, peabody developmental motor scale; SCPNT, Southern California post-rotatory nystagmus test; VOR, vestibulo-ocular reflex; y.o, years old; NOS score, Newcastle-OttawaScale*.

Figure 2 indicates the number of times each vestibular assessment was utilized among the four populations throughout the 24 studies. All studies on children with TBI assessed semicircular canal function through labyrinthine testing. Most of the studies including children with SNHL and CI also focused primarily on labyrinthine testing. Otolith and integrated balance testing, however, were also used in these populations. The sole study on children with CP assessed both otolith and labyrinthine function.

### Methodological Quality Assessment

Two pairs of reviewers obtained mean (± 1 SD) Cohen's Kappa coefficients of 0.72 ± 0.31 and 0.72 ± 0.20, indicating a “high” agreement between their scores on the Newcastle-Ottawa Scale. Final individual scores and number of stars per subsection of the Newcastle-Ottawa Scale for all 24 studies retained for analysis in this systematic review can be found in Table 1. Only one study was found to be of poor quality. Twelve studies scored between 4 and 6 stars and are therefore of moderate quality. Twelve studies scores as being of high quality.

### Analysis per Condition

Each population's characteristics, observed prevalence per condition, outcome measures, assessment tools used, and level of evidence obtained from the analysis of the studies is presented below and can be referred to in Tables 2, 3.

**Table 3:** Studies addressing central neurological conditions (Cerebral palsy and traumatic brain injury).

**References**	**Country**	**Study design**	**Participants**	**Age**	**Etiology and severity**	**Comorbidities**	**Vestibular assessment tools**	**Main results, outcomes and prevalence**	**NCO score**
Akbarfahimi et al. (44)	Iran	Case Control	Group 1: 31 children with spastic CP Group 2: 31 healthychildren	Age range: 7–12 y.o Group 1 mean age: 8.7 y.o. Group 2 mean age: 8.77y.o	Spastic CP—functional levels of I or II (GMFCS), unilateral CP (hemiplegia), bilateral spastic CP (quadriplegic and diplegic)	None specified	cVEMP (AARs)	Abnormal vestibular function was found in 15/31 (48.4%) cases through AARs or cVEMP testing. No saccular function recorded in 2/31 (6%) children through cVEMP testing.	9/9
Corwin et al. (42)	USA	Cohort, retrospective	Children post-concussion	Age range: 5–18 y.o	Concussions related to a low-impact mechanism of injury	None specified	VOMS	Abnormal VOR (gaze stability), or tandem gait was observed in 100/247 (81%) patients' post-concussion upon initial examination.	6/9
Mucha et al. (45)	USA	Cross sectional	Group 1: 64 children post-concussion Group 2: 78 healthy children	Group 1: Mean age: 13.9 y.o. Age range: 9–18 y.o Group 2: Mean age: 12.9 y.o Age range: 10–17y.o	Concussion 5.5 ± 4.0 days (range, 1–21 days) after the injury	None specified	VOMS, PCSS	Symptom provocation upon administration of the VOR item of the VOMS observed in 39/64 (61%) patients. Symptom provocation upon administration of the smooth pursuit and vertical saccade items observed in 21/64 (33%)children.	5/10
Vartiainen et al. (34)	Finland	Cohort	Group 1: 61 children treated for acute blunt head injury Group 2: 138 children who had a head injury >2 years ago and return for FU Group 3: 59 children with no head trauma to be compared to group 1 Group 4: 88 children with no head trauma to be compared to group2	Group 1: Mean age: 9.7 y.o Age range: 2–15.4 y.o. Group 2: Mean age: 12.9 y.o Age range: 5–19 y.o. Group 3: Mean age: 10.7 y.o Age range: 5–15.2 y.o Group 4: Mean age: 13 y.o. Age range: 5–19y.o	Classification of trauma: Contusion, concussions, skull fracture	None specified	Spontaneous nystagmus, positional nystagmus Pendular eye tracking test, calorictest	Subjective complaints of vertigo reported in 1/61 (2%) children of Group 1 and on 2/138 (1%) children of Group 2. Spontaneous/Positional nystagmus found on 21/46 (46%) cases of Group 1 when trauma was “acute,” and on 8/46 cases (17%) 6–12 months later. Spontaneous/Positional nystagmus found on 22/120 (18%) cases of Group 2. Canal paresis detected on 6/41 (14.6%) cases of Group 1 when trauma was “acute” and on 2/41 (4.88%) 6–12 months later through Caloric testing. Canal paresis detected on 7/113 (6%) cases of Group 2 through Caloric testing	6/9

*GMFCS, gross motor function classification system; cVEMP, cervical vestibular evoked myogenic potential; AARs, asymmetry ratio; VOMS, vestibular/ocular-motor screening; PCSS, Post-concussion symptom scale; y.o, years old; NOS score, Newcastle-Ottawa Scale*.

### CP

A single, high quality study (NCO scale: 9/9) reports that 48.4% of children (*n* = 31) aged 7 to 12 years old with spastic CP (GMFCS levels 1 and 2) exhibit a saccular dysfunction, as measured through cVEMP testing (44). The cVEMP was done with a frequency of 500 Hz air-conducted short tone burst stimuli with stimulation rate of 7.1 per second where dysfunction was categorized as an absence of the Amplitude Asymmetry Ratio.

### TBI

Three fair quality studies (NCO scale: 6/9, 6/9, and 5/10) included for this population reported vestibular dysfunction in children (*n* = 510) aged 5–19 years old with TBI (34, 42, 45). Vartiainen et al. (34) use caloric testing, from which canal paresis immediately post-injury was found in 14.6% of cases, and canal paresis 6–12 months later in 5% of cases. Moreover, when assessing for spontaneous/ positional nystagmus immediately after trauma, symptoms were seen in 46% (21/46) of children vs. 17% (8/46) 6–12 months later. The quality of this paper was scored at 6/9 on NCO Scale. Two studies performed VOMS testing (42, 45). The VOR component results show that 61 and 81% of children, in each study, respectively, demonstrated abnormal responses. Based on the NCO Scale, Mucha et al. (45) scored 5/10 and Corwin et al. (42), Vartiainen et al. (34) scored 6/9. From the three fair quality studies, the prevalence of semicircular canal dysfunction in children with TBI ranges from 14.1 to 81% when assessed up to 21 days post-trauma.

### SNHL

Twelve poor-to-high quality studies have reported the prevalence of vestibular dysfunction of children (*n* = 643) with SNHL, aged 7 months−20 years old (15, 19–21, 25, 40, 47–49, 52–54). One study scored at 3; 5 studies scored between 4 and 6; and 6 studies scored at or above 7. The data gathered suggests that a wide prevalence range of 18.75–96.1% of children experience vestibular dysfunction, independent of the etiology of SNHL. Across the studies, vestibular function was mostly assessed through vestibular-specific tests such as: Rotatory Chair Test, Caloric Test, vHIT, and VEMP. Nevertheless, the SOT scales and the balance subscales of the BOT-2 and the PDMS developmental scales were implemented in 5/12 studies.

The low-quality study by Zhou et al. (19) reported a prevalence of 91.3% for saccular dysfunction through VEMP testing. The fair quality studies (40, 47, 49, 53, 54), reported a prevalence of semi-circular canal abnormality between 5 and 58.8%. Only one fair quality study (47), reported a hyperfunction of 3%. Abnormality in otolith function was reported in 2 fair-quality studies (40, 49), to be between 17 and 33% in children with SNHL. The high-quality studies (15, 20, 21, 25, 52), reported a prevalence of vestibular dysfunction between 7.5 and 96.1%. Hyporeflexia of the vestibular system, which encompasses the terms: “areflexia,” “decreased response to…,” “decreased reaction to…,” “hypoactivity,” or “hypo-excitability,” is the most common type of dysfunction reported among eight studies, with a prevalence range within the SNHL population of 5.13–89%. Hyperreflexia of the vestibular system, which encompasses the terms: “hyperactivity” and “hyperexcitability,” is only reported in two studies. Rine et al. (20) reported it to be highly prevalent within this population, showing a percentage of 46.2%. The other study by Schwab and Kontorinis (47) reports 5% of hyperactive vestibular function in children with SNHL.

### CI

Nine fair-to-high quality studies report the prevalence of vestibular dysfunction in children (*n* = 817) who have undergone one or several unilateral or bilateral cochlear implantations, aged 1–21 years old, to be within the range of 3–84% (13, 22, 24, 25, 41, 43, 46, 50, 51). In terms of the NCO scale, 3 studies scored between 4 and 6 and 6 studies scored at or more than seven studies. Semicircular canal dysfunction post-implantation is assessed in eight studies through Caloric testing, vHIT testing, and Rotatory Chair testing, and a prevalence range of dysfunction of 8–69% is observed. Moreover, through VEMP testing, the same 8 studies report the prevalence range of otolith dysfunction to be from 19 to 84%. Solely, one study uses VNG testing and reports a prevalence of dysfunction of 3% (51).

For the fair quality studies, Wolter et al. (43), reported that prevalence for semicircular canal dysfunction was 34.5–81.8% and for otolith dysfunction was 46.1–81.8%. Thierry et al. (24) found vestibular dysfunction of 7–18.6%. For the six high quality studies (13, 22, 25, 41, 46), prevalence for semicircular canal dysfunction was 29–69% and prevalence for otolith dysfunction was 19–84%.

### Cochlear Implant or Cochlear Implantation Surgery Failure

According to Wolter et al. (43), children whose cochlear implantation failed demonstrate a larger prevalence of vestibular dysfunction. Abnormal Caloric Test responses were found in 47.3% of children with successful CIs vs. in 81.8% in children with failed implants; bilateral loss of canal function was found in 17.6% of successful implantations vs. 63.6% of failed ones; vHIT and/or high frequency rotational chair abnormal responses were found in 34.5% of successful implantations vs. 72.7% of unsuccessful ones; and bilateral abnormal VEMP responses were found in 19.4% of successful ones vs. 50% of the failures (43).

### Pre vs. Post-cochlear Implantation Comparison of Vestibular Function

Devroede et al. (41) report that the prevalence of vestibular dysfunction of children who have undergone a cochlear implantation to be of 69%, and that, after a second (contralateral) cochlear implantation, vestibular dysfunction increases by 17% (otolith dysfunction; 79 vs. 62%) and 32% (semicircular canal dysfunction). On the other hand, Licameli et al. (13) report the prevalence of otolith dysfunction to increase from 10% (2/19 abnormal responses) to 84% (16/19 abnormal responses) after the first cochlear implant whereas 26% (5/19) of the subjects' vestibular responses remained unchanged. Finally, Thierry et al. (24) report that, after the first cochlear implantation, 50% (6/12) of the children demonstrated no change in vestibular function, 33.3% (4/12) showed improvement, and 16.7% (2/12) experienced worsening through VEMP and caloric testing.

### Ipsilateral vs. Contralateral Vestibular Dysfunction

Most studies assess and report vestibular dysfunction on the side where the cochlear implantation was performed. Ipsilateral to cochlear implantation vestibular dysfunction prevalence ranges from 18.6 to 60%. However, two studies report changes in vestibular function on the contralateral side (24, 46). Cushing et al. (46) report that 23 children with unilateral CI demonstrated unilateral dysfunction of horizontal canal function on caloric testing, nine of which (40%) occurred on the non-implanted side, whereas the remaining 14 (60%) occurred on the implanted side. However, their results were not found to be statistically significant. Moreover, Thierry et al. (24) report that 18.6% of implanted children demonstrated loss of vestibular function on the side of CI, and 7% of children showed abnormal vestibular function contralateral to CI side.

## Discussion

To the best of our knowledge, this systematic review presents for the first time the prevalence of vestibular dysfunction in children across four different population groups (SNHL, CI, TBI, and CP). In the 24 analyzed studies we observed moderate-to-high level of evidence indicates a prevalence of vestibular dysfunction in the SNHL population to range between 18.5 and 96%. Moderate-to-high level of evidence indicates a prevalence of vestibular dysfunction in children with CIs to range between 3 and 84%. Likewise, a strong evidence indicates a prevalence of vestibular dysfunction in the spastic CP, GMFCS levels 1 and 2, population to be around 48%. In children with SNHL, around 18.75–96.1% of children had vestibular dysfunction. Finally, fair-to-moderate level of evidence indicates a prevalence of vestibular dysfunction in the TBI population to range between 14 and 80% immediately post-injury, and between 10 and 12% 6–8 months post TBI.

The goal of this study was to gather the prevalence within each of the conditions to further understand the need for vestibular testing in the pediatric population and see the implications these results could have on future rehabilitation. However, majority of the studies reported findings after completing individual vestibular testing of the participants and did not report an overall prevalence percentage. These percentages were calculated by were calculated by the authors from the results in the necessary studies.

In most of the studies included in this review study (i.e., 20 out of 24), a prevalence of one or more types of vestibular dysfunction in the SNHL and CI populations was observed (see Table 1). Cushing et al. (46), for instance, reported that children suffering from severe SNHL and requiring CI might exhibit canal and saccular dysfunction, which might predispose them toward poor static and dynamic balance (22). The authors also reported vestibular end organ dysfunction in almost 50% children with SNHL. They further mentioned that not only the condition but also the etiology of SNHL for instance, abnormality in cochleovestibular anatomy could help in determining children presenting a higher risk of vestibular dysfunction (22). Previously, the same research group had documented that more than a third of children with SNHL and CI exhibited vestibular dysfunctions (22).

Jafari and Asad Malayeri (48) too reported that the response threshold during VEMP was substantially lower in all the children with SNHL (P1N1 amplitude lower than controls). In addition, acoustically evoked short latency negative response was found in 40% of the children. The authors presumed that these findings might be a consequence of a response elicited from the lower parts of the brainstem and/or possibly due to the role of reflecting arch during response formation (48, 56). The importance of early vestibular assessment protocols in pediatric CI units was emphasized by Lisboa et al. (15), Raj and Gupta (21) and Wolter et al. (43). For instance, a high prevalence of peripheral vestibular syndrome was reported by Lisboa et al. (15). The authors also mentioned that these findings were independent of the etiology, gender or the grade of hearing loss. Likewise, Raj and Gupta (21) reported that one child with severe and 8 children with profound SNHL (congenital or non-syndromic) exhibited vestibular dysfunctions. Both these studies utilized caloric testing to assess vestibular dysfunctions. The authors reported that an early assessment might facilitate a better planning for surgical interventions and therefore a better prognosis for the children. Our findings are in agreement with recent review studies by Verbecque et al. (26) and Yu and Li (57) which report the higher prevalence rates of vestibular dysfunction in children with SNHL. Yu and Li (57) specifically mentioned that almost 50% patients with sudden SNHL exhibited vestibular dysfunctions due to damages at utricle-superior vestibular pathway, followed by lateral semicircular canal-superior vestibular pathway and cochlea only.

Further, Jin et al. (50) suggested that CIs might disrupt the sensory vestibular functions of the labyrinth by either resultant unilateral deafferentation or fluctuating vestibulopathy or by electrical stimulation of the vestibular system (58). The authors reported that around 50–60% of the participating children exhibited areflexia. Similarly, Jacot et al. (25) reported that almost 50% of patients with CI have vestibular dysfunctions. They also observed detrimental influence of CIs on vestibular canal and otolith function during follow-up sessions. The authors defined that the 3 months period after the CI to be a high-risk period during which vestibular impairments were prominent. Licameli et al. (13) reported the prevalence of vestibular dysfunction post-CI to be in almost 60% of children. The authors additionally mentioned that CIs also cause damage to inner ear structure i.e., fibrosis of vestibule, collapse the saccule, reduce the number of ganglion cells and affect the formation of hydrops (13, 59). Two main surgical processes were found to be mainly responsible i.e., cochleostomy and insertion of CI electrode array (60, 61). A survey also reported that a lack of consensus concerning the appropriate cochleostomy approach among CI surgeons has added toward the problem (60).

The prevalence of vestibular dysfunction was also evaluated in children with CP and TBI. However, the number of studies which had analyzed these aspects were substantially few. For instance, otoneurologic symptoms have been widely reported post head injury (62–64). Pimentel et al. (64) reported that TBI might precipitate vestibular dysfunctions because of focal lesions which might affect the labyrinth further causing unilateral vestibular hypofunction, benign paroxysmal positional vertigo and perilymphatic fistulas. A pilot study from Jury and Flynn (65) reported that 83% of young adults recovering from a TBI present with symptoms of vestibular dysfunction at some point post-trauma. Likewise, Corwin et al. (42) reported 81% prevalence for vestibular dysfunctions in children post-concussion. The authors mentioned that the widely distributed central and peripheral components of the vestibular system makes it vulnerable to the translated forces experienced during TBIs. Despite extensive studies being published on the prevalence of vestibular dysfunction due to TBI in adults (66–68), there is a substantial gap in literature concerning the pediatric population. We, in this present review, included only three studies evaluating the prevalence of vestibular dysfunction due to TBI.

Similarly, increasing evidence of vestibular deficit in children with CP have been well-discussed (69, 70), but its prevalence in children with spastic CP was only found in one study (44). Akbarfahimi et al. (44) suggested that white matter lesions, pathological changes in the cortical structure and/or deficits in afferent axons or vestibulo-spinal axons might be the predominant reasons due to which vestibular dysfunctions are experienced in children with CP (71, 72). The authors in their study used cVEMP recordings to assess saccular functions and reported a shorter amplitude in P_13_-N_13_ waves as compared to healthy controls. This reduction in amplitude might have occurred as a result of desynchronization of neural firing pattern and/or attenuation of the conductive velocity (73). Nevertheless, the lack of information regarding the incidence of vestibular dysfunction is evident, but the existence of the dysfunction has been widely reported.

### Analysis of the Included Studies

In general, small sample sizes, lack of access to follow-up examinations, comorbidities, and differences in methodology affect the accuracy of prevalence calculations. Only 7/24 studies included have a sample size larger than 100 children. Studies with larger sample sizes are usually retrospective, cohort studies. Ideally, case-control or cross-sectional studies investigating the prevalence per condition would provide a clear percentage. However, 11/24 studies included in this review are retrospective cohort studies, in which authors rely on previous information, thus old materials and methodology, and in most cases, a follow-up examination was not performed. Therefore, these excluded children are not accounted for in the prevalence percentages reported in the studies. All these factors impact the quality of the information gathered in this review. According to the subsets of the NCO scale, 52% of the studies included are of high quality, and 32% of moderate quality. Only 16% of the studies were rated with <4 points on the NCO scale but met the inclusion criteria of this review and provided further information about vestibular dysfunction in different parts of the world.

### Limitations

This systematic review looked at the prevalence of vestibular dysfunction in many neurological and neuro-developmental disabilities. It analyzed four crucial conditions to provide clinicians with an insight regarding the prevalence of vestibular dysfunction in some central, peripheral, and neurodevelopmental conditions. Also, it included all types of vestibular disorders, which allows rehabilitation professionals to generate a comprehensive conclusion about the nature of the vestibular symptoms seen in these populations. However, a few limitations persisted in this review study. Firstly, a limitation to this review is that, while screening for full text, studies stating some vestibular dysfunction, but that failed to indicate a prevalence were excluded. Also, gray literature and unpublished studies were not investigated. Secondly, this review study was not initially registered in a systematic review registry such as, PROSPERO.

### Future Directions

Several gaps in literature were identified in this systematic review. Previously published literature has compellingly mentioned a high prevalence of co-existence of vestibular dysfunctions with these conditions (67, 71, 74–76). However, a substantial deficit in literature was identified in conditions such as TBI and CP for pediatric population groups. We recommend future researchers to kindly address this substantial gap in literature. Findings from such studies might substantially enhance the capabilities of medical practitioners to preliminarily test and identify vestibular dysfunctions. In addition, the findings from the present review although reports a high prevalence of vestibular dysfunctions in SNHL and CI, but there seems to be a wide range that was identified in the studies (SNHL: 18.7–96%, CI: 3–84%). This wide range, in our opinion might be existential due to substantial heterogeneity in between the included studies. This heterogeneity could possibly be affirmed to the different testing procedures, population groups, severity of disease etc. We recommend future studies to identify a uniform, reliable, and valid battery of testing procedure that can be followed by researchers worldwide. This will not only help in easing the interpretation of the results but will also help medical practitioners to effectively design testing and rehabilitative procedures. Unfortunately, larger databases of prevalence, such as Statistics Canada, INSERM have not yet investigated the number of children affected by vestibular dysfunction, therefore increasing the importance of obtaining such knowledge in future studies would also be beneficial for patients and medical practitioners alike.

Finally, the findings of the present review suggest that a wide range of prevalence is reported in CI, SNHL and TBI populations due to differences in the testing procedures, the timing of the testing, the age of the population, the etiology, and severity of disease. Therefore, it is difficult to correlate the prevalence in specific neurodevelopmental disorders. This strongly warrants the need for further research reporting the prevalence of vestibular dysfunctions in children is required for all the populations mentioned in this review, but most importantly for the central neurological and neurodevelopmental conditions. Likewise, further studies which explore the prevalence of semicircular canal dysfunction in children with TBI, of vestibular dysfunction in children with different levels of severity of CP are also recommended. Clinicians, however, should be aware that these populations have a need for vestibular rehabilitation, and should implement the appropriate treatment interventions to optimize their rehabilitation process.

## Author Contributions

MH, AL, and ED conceptualized the study, carried out the systematic review, and wrote some parts of the paper. SG wrote the main parts of the paper. All the authors reviewed the final version of the paper.

### Conflict of Interest

The authors declare that the research was conducted in the absence of any commercial or financial relationships that could be construed as a potential conflict of interest.
