# Supplementary material for: Prevalence of Vestibular Dysfunction in Children With Neurological Disabilities: A Systematic Review
Source: Front Neurol. 2019 Dec 17;10:1294. doi: 10.3389/fneur.2019.01294 (PMC6928113; doi:10.3389/fneur.2019.01294)
Supplement: Supplementary file 1 [file Table_1.DOCX]

**Prevalence of Vestibular Dysfunction in Children with Neurological Disabilities: A systematic review**

Shashank Ghai, Mireille Hakim, Elizabeth Dannenbaum, Anouk Lamontagne,

**Supplementary File**

Table 1 Sample search strategy EMBASE

| **DATABSE** | **EMBASE** |
| --- | --- |
| **DATE** | **10/06/2019** |
| **STRATEGY** | **#1 AND #2 AND #3 AND #4 AND #5 AND #6** |
| **#1** | (‘VD’ OR ‘Vestibular disorder’ OR ‘Vestibular disorders’ OR ‘Vestibular dysfunction’ OR ‘Vestibular dysfunctions’)/de OR (VD OR Vestibular disorder OR Vestibular disorders OR Vestibular dysfunction OR Vestibular dysfunctions);ti.ab |
| **#2** | (‘CI’ OR ‘Cochlear implant’ OR ‘ABSI’ OR ‘Auditory brain stem implant’ OR ‘Cochlear prosthesis’ OR ‘Auditory prosthesis’)/de OR (CI OR Cochlear implant OR ABSI OR Auditory brain stem implant OR Cochlear prosthesis OR Auditory prosthesis);ti,ab |
| **#3** | (‘SNHL’ OR ‘Sensorineural Hearing Loss’ OR ‘Hearing loss’ OR ‘Congenital hearing loss’ OR ‘Mixed conductive sensorineural’ OR ‘Hearing loss bilateral’ OR ‘Hearing loss conductive’ OR ‘Hearing loss high frequency’ OR ‘Hearing loss functional’)/de OR (SNHL OR Sensorineural Hearing Loss OR Hearing loss OR Congenital hearing loss OR Mixed conductive sensorineural OR Hearing loss bilateral OR Hearing loss conductive OR Hearing loss high frequency OR Hearing loss functional):ti,ab |
| **#4** | ("CP" OR "Cerebral Palsy" OR "Cerebral Palsy athetoid" OR "Cerebral Palsy congenital" OR "Cerebral Palsy Diplegic infantile" OR "Cerebral Palsy dyskinetic" OR "Cerebral Palsy dystonic-rigid" OR "Cerebral Palsy hypotonic" OR "Cerebral Palsy mixed" OR "Cerebral Palsy monoplegic, infantile" OR "Cerebral Palsy quadriplegic infantile" OR "Cerebral Palsy Rolandic type" OR "Cerebral Palsy Spastic" OR "Congenital Cerebral Palsy" OR "diplegia-spastic" OR "Diplegic infantile cerebral palsy" OR "infantile cerebral palsy-diplegic" OR "infantile cerebral palsy-monoplegic" OR "infantile cerebral palsy-quadriplegic" OR "little disease" OR "little"s disease" OR "Monoplegic cerebral palsy" OR "Monoplegic infantile cerebral palsy" OR "Quadriplegic infantile cerebral palsy" OR "Spastic diplegia")/de OR (CP OR Cerebral Palsy OR Cerebral Palsy athetoid OR Cerebral Palsy congenital OR Cerebral Palsy Diplegic infantile OR Cerebral Palsy dyskinetic OR Cerebral Palsy dystonic-rigid OR Cerebral Palsy hypotonic OR Cerebral Palsy mixed OR Cerebral Palsy monoplegic, infantile OR Cerebral Palsy quadriplegic infantile OR Cerebral Palsy Rolandic type OR Cerebral Palsy Spastic OR Congenital Cerebral Palsy OR diplegia-spastic OR Diplegic infantile cerebral palsy OR infantile cerebral palsy-diplegic OR infantile cerebral palsy-monoplegic OR infantile cerebral palsy-quadriplegic OR little disease OR littles disease OR Monoplegic cerebral palsy OR Monoplegic infantile cerebral palsy OR Quadriplegic infantile cerebral palsy OR Spastic diplegia):ti,ab |
| **#5** | (‘TBI’ OR ‘TBIs’ OR ‘Traumatic brain injury’ OR ‘Traumatic brain injuries’ OR ‘Encephalopathy’ OR ‘Traumatic encephalopathy’)/de OR (TBI OR TBIs OR Traumatic brain injury OR Traumatic brain injuries OR Encephalopathy OR Traumatic encephalopathy);ti,ab |
| **#6** | (‘Analysis cross sectional’ OR ‘cross sectional study’ OR ‘cross-sectional survey’ OR ‘disease frequency surveys’ OR ‘prevalence studies’ OR ‘surveys, disease frequency’ OR ‘correlational studies’ OR ‘case control studies’ OR ‘case-base studies’ OR ‘case-comparison studies’ OR ‘case-compeer studies’ OR ‘case-referent studies’ OR ‘case-referrent studies’ OR ‘matched case-control studies’ or ‘nested case-control studies’)/de OR (Analysis cross sectional OR cross sectional study OR cross-sectional survey OR disease frequency surveys OR prevalence studies OR surveys, disease frequency OR correlational studies OR case control studies OR case-base studies OR case-comparison studies OR case-compeer studies OR case-referent studies OR case-referrent studies OR matched case-control studies or nested case-control studies);ti,ab |

Table 2 Reasons for exclusion of studies

| **Title** | **Condition** | **Reason** | **Title** | **Condition** | **Reason** |
| --- | --- | --- | --- | --- | --- |
| M. Ajalloueyan et al. [1] | CI | No prevalence reported | O. Akdogan et al. [2] | Auditory neuropathy | Other study design |
| G. Asprella-Libonati [3] | BPPV | No population of interest | C.S. Birman et al. [4] | CI | No exposure of interest |
| S.L. Cushing et al. [6] | CI | No prevalence reported | S.F. Emami and F. Farahani [8] | SNHL | No outcomes of interest |
| T.D. Fife and C. Giza [9] | CI | No population of interest | A. De Kegel et al. [7] | SNHL | No prevalence reported |
| G. Fitzgerald and C.S. Hallpike [10] | SNHL | No population of interest | P. Galaviz [11] | SNHL | No outcomes of interest |
| F. Gheysen et al. [12] | CI | No outcomes of interest | C.S. Birman et al. [5] | CI | No outcomes of interest |
| F. Goto et al. [13] | Vertigo | No exposure of interest | K.M. Guskiewicz et al. [14] | Concussion | No outcomes of interest+ No population of interest |
| K. Hamilton and A. Gelfand [15] | Pediatric migraine | No exposure of interest | O. Handzel et al. [16] | CI | No outcomes of interest |
| S.K. Hong et al. [17] | Concussion | No outcomes of interest | P.L. Huygen and P. van den Broek [18] | CI | No population of interest |
| M.A. Jury and M.C. Flynn [19] | TBI | Other study design | N.R. Keating [20] | Nystagmus | No population of interest |
| H.G. Kempf et al. [21] | CI | No outcomes of interest | J.S. Kim et al. [22] | BPPV | No population of interest |
| T. Kubo et al. [23] | CI | No population of interest | T. Kubo et al. [24] | CI | No outcomes of interest |
| R.A. Kupfer et al. [25] | SNHL | No outcomes of interest | F. Li et al. [26] | Dizziness | No population of interest |
| W. Li et al. [27] | SNHL | Vestibular assessment using non-standardized outcome measure | X. Liu and G. Li [28] | ---------- | No population of interest |
| N. Livingstone and M. McPhillips [29] | SNHL | No outcomes of interest | N. Loundon et al. [30] | CI | Vestibular assessment using non-standardized outcome measure |
| S.D. Mangeot et al. [31] | ADHD | No prevalence reported | T. Masuda and K. Kaga [32] | SNHL | No outcomes of interest |
| S. Melo Rde et al. [33] | SNHL | No outcomes of interest | R.S. Melo et al. [34] | SNHL | No prevalence reported |
| R.S. Melo et al. [35] | SNHL | No prevalence reported | A.A. Migliaccio et al. [36] | CI | No population of interest |
| L.M. Nashner et al. [37] | CP | No outcomes of interest | D.E. Newman-Toker et al. [38] | ---------- | No population of interest |
| D.E. Newman-Toker et al. [39] | Stroke | No population of interest | X. Niu et al. [40] | SNHL | No population of interest |
| R.C. O'Reilly et al. [41] | ---------- | No population of interest | A. Pajor and M. Jozefowicz-Korczynska [43] | SNHL | No population of interest |
| J.O. Phillips and D.D. Backous [44] | SNHL | Other study design | J.T. Postelmans et al. [45] | CI | Vestibular assessment using non-standardized outcome measure |
| O. Ribari et al. [46] | CI | No population of interest | R.M. Rine et al. [47] | ---------- | Other study design |
| R.M. Rine et al. [48] | SNHL | No prevalence reported | P.A. Selz et al. [49] | SNHL | No prevalence reported |
| S.B. Shum and M.Y. Pang [50] | ADHD | No prevalence reported | R.J. Smith et al. [51] | SNHL | No outcome of interest |
| H. Suarez et al. [52] | SNHL | Vestibular dysfunction as exposure | A. Szirmai et al. [53] | CI | Number of children unknown |
| C.S. Teixeira [54] | ---------- | Full text not in English or French | H.C. Tien and F.H. Linthicum, Jr. [55] | CI | No population of interest |
| I. Todt et al. [56] | N/A | No population of interest | P. Tuohimaa [57] | Concussion | Number of children unknown |
| I.O. Uysal et al. [58] | SNHL | No outcome of interest | J. Vatovec et al. [59] | DCD/CP | No population of interest |
| [60] | CI | Small population (n=3) | [61] | CI | No population of interest |
| M. Oyewumi et al. [42] | SNHL | Vestibular assessment using non-standardized outcome measure | J. Wang et al. [62] | ADHD | Full text not in English or French |
| A.C.Y. Wong and A.F. Ryan [63] | SNHL | Number of children unknown | B.Y. Wong et al. [64] | CI | Unable to find article |
| O. Zagolski [66] | ---------- | Full text not in English or French | J. Wu et al. [65] | SNHL | Full text not in English or French |
| M.A. Zare et al. [68] | Concussion | No outcome of interest | Y.-F. Zang et al. [67] | ADHD | Number of children unknown |

Table 3 PRISMA Checklist (From: Moher D, Liberati A, Tetzlaff J, Altman DG, The PRISMA Group (2009). Preferred Reporting Items for Systematic Reviews and Meta-Analyses: The PRISMA Statement. PLoS Med 6(6): e1000097. doi:10.1371/journal.pmed1000097)

| **Section/topic** | **#** | | **Checklist item** | | | **Reported on page #** | |
| --- | --- | --- | --- | --- | --- | --- | --- |
| **TITLE** | | | | | |  | |
| Title | 1 | | Identify the report as a systematic review, meta-analysis, or both. | | | 1 | |
| **ABSTRACT** | | | | | |  | |
| Structured summary | 2 | | Provide a structured summary including, as applicable: background; objectives; data sources; study eligibility criteria, participants, and interventions; study appraisal and synthesis methods; results; limitations; conclusions and implications of key findings; systematic review registration number. | | | 1-2 | |
| **INTRODUCTION** | | | | | |  | |
| Rationale | 3 | | Describe the rationale for the review in the context of what is already known. | | | 3-6 | |
| Objectives | 4 | | Provide an explicit statement of questions being addressed. | | | 6 | |
| **METHODS** | | | | | |  | |
| Protocol and registration | 5 | | Indicate if a review protocol exists, if and where it can be accessed (e.g., Web address), and, if available, provide registration information including registration number. | | | - | |
| Eligibility criteria | 6 | | Specify study characteristics (e.g., PICOS, length of follow-up) and report characteristics (e.g., years considered, language, publication status) used as criteria for eligibility, giving rationale. | | | 7 | |
| Information sources | 7 | | Describe all information sources (e.g., databases with dates of coverage, contact with study authors to identify additional studies) in the search and date last searched. | | | 7 | |
| Search | 8 | | Present full electronic search strategy for at least one database, including any limits used, such that it could be repeated. | | | Supplementary table 1 | |
| Study selection | 9 | | State the process for selecting studies (i.e., screening, eligibility, included in systematic review, and, if applicable, included in the meta-analysis). | | | 6-7, Figure 1 | |
| Data collection process | 10 | | Describe method of data extraction from reports (e.g., piloted forms, independently, in duplicate) and any processes for obtaining and confirming data from investigators. | | | Supplementary file form 1 | |
| Data items | 11 | | List and define all variables for which data were sought (e.g., PICOS, funding sources) and any assumptions and simplifications made. | | | Supplementary table 1 | |
| Risk of bias in individual studies | 12 | | Describe methods used for assessing risk of bias of individual studies (including specification of whether this was done at the study or outcome level), and how this information is to be used in any data synthesis. | | | 8, 10-15, Table 1 | |
| Summary measures | 13 | | State the principal summary measures (e.g., risk ratio, difference in means). | | | 10-15, Table 2-4 | |
| Synthesis of results | 14 | | Describe the methods of handling data and combining results of studies, if done, including measures of consistency (e.g., I^2^) for each meta-analysis. | | | - | |
| **Section/topic** | | **#** | | **Checklist item** | **Reported on page #** | |  |
| Risk of bias across studies | | 15 | | Specify any assessment of risk of bias that may affect the cumulative evidence (e.g., publication bias, selective reporting within studies). | 8, 10-15 | |  |
| Additional analyses | | 16 | | Describe methods of additional analyses (e.g., sensitivity or subgroup analyses, meta-regression), if done, indicating which were pre-specified. | - | |  |
| **RESULTS** | | | | |  | |  |
| Study selection | | 17 | | Give numbers of studies screened, assessed for eligibility, and included in the review, with reasons for exclusions at each stage, ideally with a flow diagram. | Figure 1, Supplementary Table 2 | |  |
| Study characteristics | | 18 | | For each study, present characteristics for which data were extracted (e.g., study size, PICOS, follow-up period) and provide the citations. | 8-9, Table 2 | |  |
| Risk of bias within studies | | 19 | | Present data on risk of bias of each study and, if available, any outcome level assessment (see item 12). | 8, Table 1 | |  |
| Results of individual studies | | 20 | | For all outcomes considered (benefits or harms), present, for each study: (a) simple summary data for each intervention group (b) effect estimates and confidence intervals, ideally with a forest plot. | 10-15, Table 2-4 | |  |
| Synthesis of results | | 21 | | Present results of each meta-analysis done, including confidence intervals and measures of consistency. | - | |  |
| Risk of bias across studies | | 22 | | Present results of any assessment of risk of bias across studies (see Item 15). | 10-15, Table 1 | |  |
| Additional analysis | | 23 | | Give results of additional analyses, if done (e.g., sensitivity or subgroup analyses, meta-regression [see Item 16]). | - | |  |
| **DISCUSSION** | | | | |  | |  |
| Summary of evidence | | 24 | | Summarize the main findings including the strength of evidence for each main outcome; consider their relevance to key groups (e.g., healthcare providers, users, and policy makers). | 15-19 | |  |
| Limitations | | 25 | | Discuss limitations at study and outcome level (e.g., risk of bias), and at review-level (e.g., incomplete retrieval of identified research, reporting bias). | 20 | |  |
| Conclusions | | 26 | | Provide a general interpretation of the results in the context of other evidence, and implications for future research. | 15, 21-22 | |  |
| **FUNDING** | | | | |  | |  |
| Funding | | 27 | | Describe sources of funding for the systematic review and other support (e.g., supply of data); role of funders for the systematic review. | - | |  |

For more information, visit: **www.prisma-statement.org**.

**Form 1 Data Extraction Form**

| **General Descriptive Information** | | | | |  |
| --- | --- | --- | --- | --- | --- |
| **Authors** |  | | **Year** |  |  |
| **Title** |  | | | |  |
| **Journal** |  | | | |  |
| **Country** |  | | | |  |
| **Objectives** |  | | | |  |
| **Methods** | | | | |  |
| **Study design** |  | | **Population of interest** |  |  |
| **Inclusion criteria** |  | | **Exclusion criteria** |  |  |
| **Control group** |  | | **Sample size** |  |  |
| **Outcomes Assessed** |  | | **Outcome assessment tools** |  |  |
| **Characteristics of the population of interest** | | | | |  |
| **Sample size** |  | | **Ethnicity** |  |  |
| **Age** |  | | **Severity/ etiology of disorder** |  |  |
| **Gender** |  | | **Comorbidities** |  |  |
| **Results** | | | | |  |
| **Overview:** | **Need to contact authors for clarification? Y/N**  **Author information provided? Y/N** | | | |  |
| **Prevalence** |  | | | |  |
| **Discussion** |  | | | |  |
| **Newcastle Ottawa scale** | Section | Comparability | Exposure | Strength of statement |  |
|  |  |  |  |  |  |
| **Paper Limitation** | **Possible biases**: selection bias, information bias, performance bias, confounding bias. | | | |  |

**References**

[1] M. Ajalloueyan, M. Saeedi, M. Sadeghi and F.Z. Abdollahi, The effects of cochlear implantation on vestibular function in 1–4 years old children, *International journal of pediatric otorhinolaryngology* **94** (2017), 100-103.

[2] O. Akdogan, A. Selcuk, I. Ozcan and H. Dere, Vestibular nerve functions in children with auditory neuropathy, *International journal of pediatric otorhinolaryngology* **72** (2008), 415-419.

[3] G. Asprella-Libonati, Lateral canal BPPV with pseudo-spontaneous nystagmus masquerading as vestibular neuritis in acute vertigo: a series of 273 cases, *Journal of Vestibular Research* **24** (2014), 343-349.

[4] C.S. Birman, J.A. Brew, W.P.R. Gibson and E.J. Elliott, CHARGE syndrome and Cochlear implantation: difficulties and outcomes in the paediatric population, *International journal of pediatric otorhinolaryngology* **79** (2015), 487-492.

[5] C.S. Birman, W.P. Gibson and E.J. Elliott, Pediatric cochlear implantation: associated with minimal postoperative pain and dizziness, *Otol Neurotol* **36** (2015), 220-222.

[6] S.L. Cushing, R. Chia, A.L. James, B.C. Papsin and K.A. Gordon, A test of static and dynamic balance function in children with cochlear implants: the vestibular olympics, *Archives of Otolaryngology–Head & Neck Surgery* **134** (2008), 34-38.

[7] A. De Kegel, L. Maes, T. Baetens, I. Dhooge and H. Van Waelvelde, The influence of a vestibular dysfunction on the motor development of hearing-impaired children, *Laryngoscope* **122** (2012), 2837-2843.

[8] S.F. Emami and F. Farahani, Saccular dysfunction in children with sensorineural hearing loss and auditory neuropathy/auditory dys-synchrony, *Acta Otolaryngol* **135** (2015), 1298-1303.

[9] T.D. Fife and C. Giza, Posttraumatic vertigo and dizziness, *Seminars in Neurology* **33** (2013), 238-243.

[10] G. Fitzgerald and C.S. Hallpike, Studies in human vestibular function: I. Observations on the directional preponderance (“Nystagmusbereitschaft”) of caloric nystagmus resulting from cerebral lesions, *Brain* **65** (1942), 115-137.

[11] P. Galaviz, Vestibular Function and Motor Proficiency of Children with Impaired Hearing, or with Learning Disability and Motor Impairments, *Pediatric Physical Therapy* **2** (1990), 111-112.

[12] F. Gheysen, G. Loots and H. Van Waelvelde, Motor development of deaf children with and without cochlear implants, *Journal of Deaf Studies and Deaf Education* **13** (2007), 215-224.

[13] F. Goto, N. Suzuki, M. Hara, N. Tsuchihashi and N. Morimoto, A retrospective series of 77 pediatric patients with vertigo at a national center for child health and development, *Nihon Jibiinkoka Gakkai kaiho* **118** (2015), 860-866.

[14] K.M. Guskiewicz, S.E. Ross and S.W. Marshall, Postural Stability and Neuropsychological Deficits After Concussion in Collegiate Athletes, *J Athl Train* **36** (2001), 263-273.

[15] K. Hamilton and A. Gelfand, Frequency of vertigo in pediatric migraine, *Cephalalgia* **37 (1 Supplement 1)** (2017), 271-272.

[16] O. Handzel, B.J. Burgess and J.B. Nadol, Jr., Histopathology of the peripheral vestibular system after cochlear implantation in the human, *Otol Neurotol* **27** (2006), 57-64.

[17] S.K. Hong, J.H. Kim, H.J. Kim and H.J. Lee, Changes in the gray matter volume during compensation after vestibular neuritis: a longitudinal VBM study, *Restor Neurol Neurosci* **32** (2014), 663-673.

[18] P.L. Huygen and P. van den Broek, Vestibular function pre- and post-cochlear implantation, *J Otolaryngol* **24** (1995), 262.

[19] M.A. Jury and M.C. Flynn, Auditory and vestibular sequelae to traumatic brain injury: a pilot study, *N Z Med J* **114** (2001), 286-288.

[20] N.R. Keating, A comparison of duration of nystagmus as measured by the Southern California Postrotary Nystagmus Test and electronystagmography, *Am J Occup Ther* **33** (1979), 92-97.

[21] H.G. Kempf, K. Johann and T. Lenarz, Complications in pediatric cochlear implant surgery, *Eur Arch Otorhinolaryngol* **256** (1999), 128-132.

[22] J.S. Kim, S.Y. Oh, S.H. Lee, J.H. Kang, D.U. Kim, S.H. Jeong, K.D. Choi, I.S. Moon, B.K. Kim, H.J. Oh and H.J. Kim, Randomized clinical trial for apogeotropic horizontal canal benign paroxysmal positional vertigo, *Neurology* **78** (2012), 159-166.

[23] T. Kubo, K. Yamamoto, T. Iwaki, K. Doi and M. Tamura, Different forms of dizziness occurring after cochlear implant, *Eur Arch Otorhinolaryngol* **258** (2001), 9-12.

[24] T. Kubo, K. Yamamoto, T. Iwaki and A. Sugii, Comparison of hearing ability in cochlear implant subjects with normal and ossified cochlea, *Adv Otorhinolaryngol* **57** (2000), 331-334.

[25] R.A. Kupfer, R.C. Hoesli, G.E. Green and M.C. Thorne, The relationship between jugular bulb-vestibular aqueduct dehiscence and hearing loss in pediatric patients, *Otolaryngol Head Neck Surg* **146** (2012), 473-477.

[26] F. Li, J. Zhuang, Y. Chen, B. Gao, H. Gu and X. Zhou, [Difference of cervical vestibular evoked myogenic potentials in different audition stage of Meniere disease], *Lin Chung Er Bi Yan Hou Tou Jing Wai Ke Za Zhi* **30** (2016), 9-12.

[27] W. Li, J. Qiu, D. Yu and Y. Zeng, [A questionnaires study on cochlear implantation in patients with white matter changes], *Lin Chung Er Bi Yan Hou Tou Jing Wai Ke Za Zhi* **28** (2014), 1205-1207.

[28] X. Liu and G. Li, [Clinical study of benign paroxysmal positional vertigo recurrence], *Lin Chuang Er Bi Yan Hou Tou Jing Wai Ke Za Zhi = Journal Of Clinical Otorhinolaryngology, Head, & Neck Surgery* **23** (2009), 304-306.

[29] N. Livingstone and M. McPhillips, Motor skill deficits in children with partial hearing, *Dev Med Child Neurol* **53** (2011), 836-842.

[30] N. Loundon, M. Blanchard, G. Roger, F. Denoyelle and E.N. Garabedian, Medical and surgical complications in pediatric cochlear implantation, *Archives of Otolaryngology–Head & Neck Surgery* **136** (2010), 12-15.

[31] S.D. Mangeot, L.J. Miller, D.N. McIntosh, J. McGrath-Clarke, J. Simon, R.J. Hagerman and E. Goldson, Sensory modulation dysfunction in children with attention-deficit-hyperactivity disorder, *Dev Med Child Neurol* **43** (2001), 399-406.

[32] T. Masuda and K. Kaga, Relationship between acquisition of motor function and vestibular function in children with bilateral severe hearing loss, *Acta Otolaryngol* **134** (2014), 672-678.

[33] S. Melo Rde, A. Lemos, C.F. Macky, M.C. Raposo and K.M. Ferraz, Postural control assessment in students with normal hearing and sensorineural hearing loss, *Braz J Otorhinolaryngol* **81** (2015), 431-438.

[34] R.S. Melo, A. Lemos, M.C.F. Raposo, R.B. Belian and K.M. Ferraz, Balance performance of children and adolescents with sensorineural hearing loss: Repercussions of hearing loss degrees and etiological factors, *Int J Pediatr Otorhinolaryngol* **110** (2018), 16-21.

[35] R.S. Melo, S. Marinho, M.E.A. Freire, R.A. Souza, H.A.M. Damasceno and M.C.F. Raposo, Static and dynamic balance of children and adolescents with sensorineural hearing loss, *Einstein (Sao Paulo)* **15** (2017), 262-268.

[36] A.A. Migliaccio, C.C. Della Santina, J.P. Carey, J.K. Niparko and L.B. Minor, The vestibulo-ocular reflex response to head impulses rarely decreases after cochlear implantation, *Otol Neurotol* **26** (2005), 655-660.

[37] L.M. Nashner, A. Shumway-Cook and O. Marin, Stance posture control in select groups of children with cerebral palsy: deficits in sensory organization and muscular coordination, *Exp Brain Res* **49** (1983), 393-409.

[38] D.E. Newman-Toker, C.A. Camargo, Jr., Y.H. Hsieh, A.J. Pelletier and J.A. Edlow, Disconnect between charted vestibular diagnoses and emergency department management decisions: a cross-sectional analysis from a nationally representative sample, *Acad Emerg Med* **16** (2009), 970-977.

[39] D.E. Newman-Toker, K.A. Kerber, Y.H. Hsieh, J.H. Pula, R. Omron, A.S. Saber Tehrani, G. Mantokoudis, D.F. Hanley, D.S. Zee and J.C. Kattah, HINTS outperforms ABCD2 to screen for stroke in acute continuous vertigo and dizziness, *Acad Emerg Med* **20** (2013), 986-996.

[40] X. Niu, Y. Zhang, Q. Zhang, X. Xu, P. Han, Y. Cheng, Y. Gao, R. Zhang, Y. Yang, Z. Chen, J. Hu, Y. Chen and M. Xu, The relationship between hearing loss and vestibular dysfunction in patients with sudden sensorineural hearing loss, *Acta Otolaryngol* **136** (2016), 225-231.

[41] R.C. O'Reilly, T. Morlet, B.D. Nicholas, G. Josephson, D. Horlbeck, L. Lundy and A. Mercado, Prevalence of vestibular and balance disorders in children, *Otol Neurotol* **31** (2010), 1441-1444.

[42] M. Oyewumi, N.E. Wolter, E. Heon, K.A. Gordon, B.C. Papsin and S.L. Cushing, Using balance function to screen for vestibular impairment in children with sensorineural hearing loss and cochlear implants, *Otology & Neurotology* **37** (2016), 926-932.

[43] A. Pajor and M. Jozefowicz-Korczynska, Prognostic factors for vestibular impairment in sensorineural hearing loss, *Eur Arch Otorhinolaryngol* **265** (2008), 403-407.

[44] J.O. Phillips and D.D. Backous, Evaluation of vestibular function in young children, *Otolaryngol Clin North Am* **35** (2002), 765-790.

[45] J.T. Postelmans, B. Cleffken and R.J. Stokroos, Post-operative complications of cochlear implantation in adults and children: five years' experience in Maastricht, *J Laryngol Otol* **121** (2007), 318-323.

[46] O. Ribari, M. Kustel, A. Szirmai and G. Repassy, Cochlear implantation influences contralateral hearing and vestibular responsiveness, *Acta Otolaryngol* **119** (1999), 225-228.

[47] R.M. Rine, E. Dannenbaum and J. Szabo, 2015 Section on Pediatrics Knowledge Translation Lecture: Pediatric Vestibular-Related Impairments, *Pediatr Phys Ther* **28** (2016), 2-6.

[48] R.M. Rine, S. Lindeblad, P. Donovan, K. Vergara, J. Gostin and K. Mattson, Balance and Motor Skills in Young Children With Sensorineural Hearing Impairment: A Preliminary Study, **8** (1996), 55-61.

[49] P.A. Selz, M. Girardi, H.R. Konrad and L.F. Hughes, Vestibular deficits in deaf children, *Otolaryngol Head Neck Surg* **115** (1996), 70-77.

[50] S.B. Shum and M.Y. Pang, Children with attention deficit hyperactivity disorder have impaired balance function: involvement of somatosensory, visual, and vestibular systems, *J Pediatr* **155** (2009), 245-249.

[51] R.J. Smith, J.F. Bale, Jr. and K.R. White, Sensorineural hearing loss in children, *Lancet* **365** (2005), 879-890.

[52] H. Suarez, S. Angeli, A. Suarez, B. Rosales, X. Carrera and R. Alonso, Balance sensory organization in children with profound hearing loss and cochlear implants, *Int J Pediatr Otorhinolaryngol* **71** (2007), 629-637.

[53] A. Szirmai, O. Ribari and G. Repassy, Air caloric computer system application in monitoring vestibular function changes after cochlear implantation, *Otolaryngol Head Neck Surg* **125** (2001), 631-634.

[54] C.S. Teixeira, Hidroginástica na reabilitação vestibular para idosos com queixas de tontura, (2008).

[55] H.C. Tien and F.H. Linthicum, Jr., Histopathologic changes in the vestibule after cochlear implantation, *Otolaryngol Head Neck Surg* **127** (2002), 260-264.

[56] I. Todt, D. Basta and A. Ernst, Does the surgical approach in cochlear implantation influence the occurrence of postoperative vertigo?, *Otolaryngol Head Neck Surg* **138** (2008), 8-12.

[57] P. Tuohimaa, Vestibular disturbances after acute mild head injury, *Acta Otolaryngol Suppl* **359** (1978), 3-67.

[58] I.O. Uysal, T. Muderris, K. Polat, S. Yuce and S. Gulturk, Is the time from the onset to the treatment a prognostic indicator for hearing recovery in idiopathic sudden sensorineural hearing loss?, *Kulak Burun Bogaz Ihtis Derg* **25** (2015), 70-76.

[59] J. Vatovec, M. Velikovic, L. Smid, K. Brenk and M. Zargi, Impairments of vestibular system in infants at risk of early brain damage, *Scand Audiol Suppl* (2001), 191-193.

[60] D. Vibert, R. Hausler, M. Kompis and M. Vischer, Vestibular function in patients with cochlear implantation, *Acta Otolaryngol Suppl* **545** (2001), 29-34.

[61] M. Viccaro, P. Mancini, R. La Gamma, E. De Seta, E. Covelli and R. Filipo, Positional vertigo and cochlear implantation, *Otol Neurotol* **28** (2007), 764-767.

[62] J. Wang, Y. Wang and Y. Ren, [A case-control study on balance function of attention deficit hyperactivity disorder (ADHD) children], *Beijing Da Xue Xue Bao Yi Xue Ban* **35** (2003), 280-283.

[63] A.C.Y. Wong and A.F. Ryan, Mechanisms of sensorineural cell damage, death and survival in the cochlea, *Frontiers in aging neuroscience* **7** (2015), 58-58.

[64] B.Y. Wong, Y. Hui, D. Au and W. Wei, Economic evaluation of cochlear implantation, *Adv Otorhinolaryngol* **57** (2000), 377-381.

[65] J. Wu, J. Wang, J. Xie, L. Han and L. Gao, [Clinical characterization of audiometric and vestibular evoked myogenic potentials in patients with large vestibular aqueduct syndrome], *Lin Chung Er Bi Yan Hou Tou Jing Wai Ke Za Zhi* **24** (2010), 25-27.

[66] O. Zagolski, [Vestibular tests in infants with TORCH and after CNS infections], *Przegl Lek* **62** (2005), 769-771.

[67] Y.-F. Zang, B. Gu, Q. Qian and Y. Wang, Objective measurement of the balance dysfunction in Attention Deficit Hyperactivity Disorder in Children, *Chinese Journal of Clinical Rehabilitation* **6** (2002).

[68] M.A. Zare, K. Ahmadi, S.A. Zadegan, D. Farsi and V. Rahimi-Movaghar, Effects of brain contusion on mild traumatic brain-injured patients, *Int J Neurosci* **123** (2013), 65-69.
